# Late renal toxicity of treatment for childhood malignancy: risk factors, long-term outcomes, and surveillance

**DOI:** 10.1007/s00467-017-3662-z

**Published:** 2017-04-22

**Authors:** Roderick Skinner

**Affiliations:** Department of Paediatric and Adolescent Haematology/Oncology, and Children’s Haemopoietic Stem Cell Transplant Unit, Great North Children’s Hospital, Newcastle upon Tyne, NE1 4LP UK

**Keywords:** Nephrotoxicity, Glomerular toxicity, Renal tubular toxicity, Ifosfamide, Platinum agents, Nephrectomy, Renal radiotherapy

## Abstract

Chronic glomerular and tubular nephrotoxicity is reported in 20–50% and 20–25%, respectively, of children and adolescents treated with ifosfamide and 60–80% and 10–30%, respectively, of those given cisplatin. Up to 20% of children display evidence of chronic glomerular damage after unilateral nephrectomy for a renal tumour. Overall, childhood cancer survivors have a ninefold higher risk of developing renal failure compared with their siblings. Such chronic nephrotoxicity may have multiple causes, including chemotherapy, radiotherapy exposure to kidneys, renal surgery, supportive care drugs and tumour-related factors. These cause a wide range of chronic glomerular and tubular toxicities, often with potentially severe clinical sequelae. Many risk factors for developing nephrotoxicity, mostly patient and treatment related, have been described, but we remain unable to predict all episodes of renal damage. This implies that other factors may be involved, such as genetic polymorphisms influencing drug metabolism. Although our knowledge of the long-term outcomes of chronic nephrotoxicity is increasing, there is still much to learn, including how we can optimally predict or achieve early detection of nephrotoxicity. Greater understanding of the pathogenesis of nephrotoxicity is needed before its occurrence can be prevented.

## Introduction

Paediatric and adult nephrologists and oncologists involved in long-term follow-up of childhood cancer survivors (CCS) encounter many patients with chronic glomerular and/or renal tubular impairment. Balancing the long-term risks and benefits of potentially nephrotoxic treatments is tricky, and progress in preventing nephrotoxicity remains frustratingly out of reach. In addition, it remains difficult to achieve accurate early recognition, let alone prediction, of incipient significant renal dysfunction that would potentially allow treatment modification early enough to avoid chronic nephrotoxicity. A report from the Childhood Cancer Survivor Study of >10,000 CCS treated in the 1970s and 1980s reported that 0.5% had developed renal failure or were requiring dialysis by a mean age of 27 years (18 years from initial cancer diagnosis), representing a ninefold increased risk compared with their siblings [1]. Although modern treatment protocols have been designed with the intention of reducing chronic renal toxicity, the greater use of potentially nephrotoxic chemotherapy since the 1970s and the ever-increasing intensity of treatment regimens for many diagnoses, implies that chronic nephrotoxicity will probably be at least as prevalent in contemporary CCS cohorts.

The causes of such chronic renal damage in CCS are varied. Occasionally, malignant disease itself may cause chronic renal impairment, for example, by damaging normal renal tissue by tumour infiltration, or long-term sequelae of urinary tract obstruction or tumour lysis syndrome. There are many treatment-related causes for chronic renal damage in CCS, including chemotherapy (most commonly cisplatin or ifosfamide), radiotherapy, surgery, immunotherapy and supportive treatment (aminoglycoside antibiotics, amphotericin). The kidneys’ excretory function relies on high renal blood flow across a large glomerular endothelial surface area followed by extremely active tubular reabsorption and secretion, but these normal physiological processes expose renal cells to toxic substances that may accumulate or undergo further intracellular metabolism. It is therefore not surprising that the kidneys are highly vulnerable to damaging adverse effects from a variety of drugs as they undergo renal excretion and metabolism [2]. The reliance of kidney function on complex vascular structures and metabolically active cells renders renal tissue very sensitive to radiotherapy. Chronic radiation nephropathy may present with proteinuria, hypertension and reduced glomerular filtration rate (GFR), which may be progressive, and was observed in 46% of adults who received 20 Gy radiotherapy exposing the left kidney during treatment for peptic ulcer disease [3, 4]. The severity of chronic kidney disease (CKD) appears to be related to dose and treatment volume, and dose–volume constraints have been recommended based on an estimated risk for chronic nephrotoxicity of <5% [3]. Renal haemodynamics may be significantly disturbed by direct destruction or removal of large amounts of renal tissue—as in renal tumours or infiltration—or surgery for renal tumours leading to diminished glomerular filtration surface area and hence reduced GFR, or hyperfiltration across the remaining glomeruli, or a mixture of both. Glomerular hyperfiltration is well documented as a long-term consequence of nephrectomy [5], whilst case reports in CCS have described proteinuria, hypertension and progressive CKD due to focal glomerulosclerosis, most likely as a consequence of hyperfiltration [6]. A recent single-centre study revealed that of 35 adult-aged, long-term (≥5 years) survivors of childhood nonsyndromic unilateral renal tumours treated by unilateral nephrectomy, chemotherapy (in 31 survivors) and radiotherapy (in 8), 23% had a mildly reduced GFR (60–89 ml/min/1.73m^2^), 9% chronic albuminuria and 3% hypertension [7]. It is important to recognise that the consequences of nephrotoxicity are not limited to the direct sequelae of renal impairment. Significant glomerular dysfunction may limit further chemotherapy options available to the patient during both first-line and subsequent relapse treatment and may ultimately have an adverse effect on the patient’s outcome by preventing use of optimum chemotherapy agents and schedules.

This educational review summarises what we know and what we are still learning about these important issues, with particular emphasis on nephrotoxicity due to ifosfamide and platinum agents (cisplatin and carboplatin), since these remain the most frequently encountered drug-related causes of chronic renal impairment in CCS. It also outlines clinical characteristics, risk factors and management of ifosfamide and platinum-induced nephrotoxicity and describes emerging information on long-term outcomes. It then reflects on our still incomplete knowledge of why nephrotoxicity occurs and how we can best detect it at an early and potentially modifiable stage, or even ideally prevent it happening at all. Finally, it highlights the lack of knowledge about the potential for chronic nephrotoxicity in patients treated with the emerging generation of anticancer drugs and the very-long-term (i.e. ≥20 years) outcome of chronic renal damage in CCS.

## What do we already know?

### Clinical features of chronic nephrotoxicity

#### Ifosfamide

Ifosfamide may cause both acute and chronic glomerular and tubular damage. Acute glomerular toxicity manifesting as acute kidney injury (AKI) is uncommon in children but well recognised in adults [8]. Renal function may not recover fully, leading to CKD, or the latter may occur even in the absence of a previous episode of AKI [9, 10]. Stages 2 and 3 CKD have been reported in 20–50% of children and adolescents after completion of ifosfamide treatment [11, 12]. Acute proximal tubular toxicity occurs in 20–25% of children given ifosfamide, typically leading to hypophosphataemia due to phosphaturia [12]. If prolonged, hypophosphataemic rickets (HR) [13] may ensue, or osteomalacia in adults [14]. More detailed evaluation usually reveals renal glycosuria (in the absence of hyperglycaemia) and aminoaciduria, whilst proximal renal tubular acidosis (RTA) may be identified and—in severe cases—a generalised proximal tubular reabsorptive impairment (Fanconi syndrome) [12, 13]. Although reported less commonly, significant distal nephron impairment may lead to nephrogenic diabetes insipidus, resulting in severe polyuria, and distal RTA [13]. These acute toxicities may complicate delivery of anticancer treatment and often persist for years after treatment completion, resulting in long-term electrolyte and mineral supplementation orally. However, more recent long-term follow-up studies have suggested that tubular toxicity improves over a period of several years [15], although similar recovery does not appear to occur in glomerular function. Significant chronic ifosfamide nephrotoxicity appears to be common in adults, with 45% of 217 1-year survivors and 53% of 154 5-year survivors with CKD stage ≥3 in a large cohort study [16]. Additional features of chronic nephrotoxicity include hypertension (although this appears to be uncommon) and growth impairment due to HR [13, 17].

#### Platinum agents

##### Cisplatin

Likewise, cisplatin may also cause both acute and chronic glomerular and tubular toxicity. Compared with ifosfamide, there are more reports, both in case series and individual case reports, of AKI and subsequent CKD due to cisplatin [18–20]. The frequency of CKD with reduced GFR (stage ≥2) is reported to be 60–80% in children treated with cisplatin [12, 13]. In contrast to that associated with ifosfamide, cisplatin-induced tubular damage leads to magnesuria and hence chronic hypomagnesaemia, which is reported in 10–30% of children treated with cisplatin [18, 19, 21]. Hypocalcaemia may occur less commonly and usually appears to be secondary to hypomagnesaemia [22]. More subtle distal nephron damage is described, resulting in the association of hypocalciuria and hypokalaemic metabolic alkalosis, as well as polyuria, the consequences of which are seldom clinically significant [23]. Thrombotic microangiopathy (TMA), sometimes described as haemolyic uraemic syndrome (HUS) in older literature, may cause AKI after cisplatin treatment [24]—although it is more commonly seen in adults—following treatment with mitomycin, gemcitabine or targeted anticancer agents [25]. Chronic glomerular damage is also common in adults, with a large cohort study reporting stage 3 CKD in 29% of 533 1-year survivors and 33% of 3975-year survivors treated with cisplatin [26]. Hypertension is also well described and may be due to vascular or renal toxicity or both [27].

##### Carboplatin

Carboplatin nephrotoxicity is similar in nature in terms of causing glomerular impairment and hypomagnesaemia but less common (especially for glomerular toxicity) and usually much less severe than cisplatin-induced renal toxicity [21, 28].

### Risk factors

Knowledge of risk factors for the development of treatment-associated acute and chronic nephrotoxicity in children with cancer is clearly vital in the clinical care of individual patients. It also facilitates the design of future treatment protocols by improving understanding about the relative likelihood of efficacy and risk of toxicity and allowing more informed use of potentially harmful treatments. Agents in current use likely to cause nephrotoxicity are also highly effective at treating cancer, so their continued use remains necessary to maximise the chances of cure for as many children as possible. As for many widely used contemporary cytotoxic treatments for childhood malignancy, it remains important to learn how to use the existing potentially toxic treatments more safely until we find better alternatives [29].

In general, risk factors may be patient (e.g. age at treatment, previous history of renal disease, toxicity) or treatment related. Timing of assessment may also be a factor where the likelihood and/or severity of toxicity is characterised by deterioration (e.g. in chronic radiation nephropathy [3, 4]) or improvement (e.g. ifosfamide-induced tubular toxicity [15]). Treatment-related factors differ according to the treatment in question but may include drug (or radiation) dose (both individual and cumulative doses), dose schedule and intensity (i.e. how much over a specific period of time) and pharmacological parameters (e.g. a drug’s pharmacokinetic profile). However, there is increasing recognition that these traditional risk factors fail to explain all episodes of renal toxicity. Indeed, although poorly understood in the context of nephrotoxicity, there is emerging interest in the study of pharmacogenetics whereby patient and treatment factors may interact in some individual patients with increased vulnerability due to particular genetic polymorphisms affecting, for example, drug metabolism or renal tubular excretion, thus increasing the risk of toxicity even after low treatment doses [30].

#### Ifosfamide

Treatment-related risk factors for ifosfamide nephrotoxicity are well established and include a high cumulative ifosfamide dose [10, 12, 31], previous or concurrent treatment with cisplatin and prior nephrectomy [32]. Young age at treatment also appears to be relevant [10], although its importance as a predictor of toxicity remains uncertain, with some studies reporting no effect independent of cumulative ifosfamide dose. Clinical experience suggests, and several studies appear to show, an increased risk of nephrotoxicity in young children [10, 31, 33, 34], although others have not confirmed this observation [12, 32]. Most published reports of severe toxicity are in infants and young children, a population highly vulnerable to proximal tubular toxicity and its consequences, such as growth impairment [13]. Nevertheless, there remains uncertainty about the role of confounding factors, such as cumulative ifosfamide dose and additional cisplatin treatment. Furthermore, some large medium-term studies do not show an independent effect of young age [12, 32], whilst very-long-term studies show either no [15] or only a weak effect (relative risk 1.08) of older age at treatment [35].

Although poorly documented in published studies, the importance of pre-existing renal impairment is widely recognised in clinical practice and consistent with the known adverse impact of prior nephrectomy [32]. In contrast to initial hopes, there is no evidence that the ifosfamide administration infusion duration (bolus, short or prolonged infusion), nor the drug’s pharmacokinetic profile, influence long-term nephrotoxicity [36]. Although currently known risk factors fail to predict all episodes of chronic ifosfamide nephrotoxicity, avoidance of higher cumulative ifosfamide doses may contribute to a reduction in their frequency and severity. However, although lower doses are associated with less acute tubular toxicity [37], no randomised clinical trial or comparative longitudinal epidemiological data is available to confirm that long-term toxicity is reduced.

**Fig. 1:**
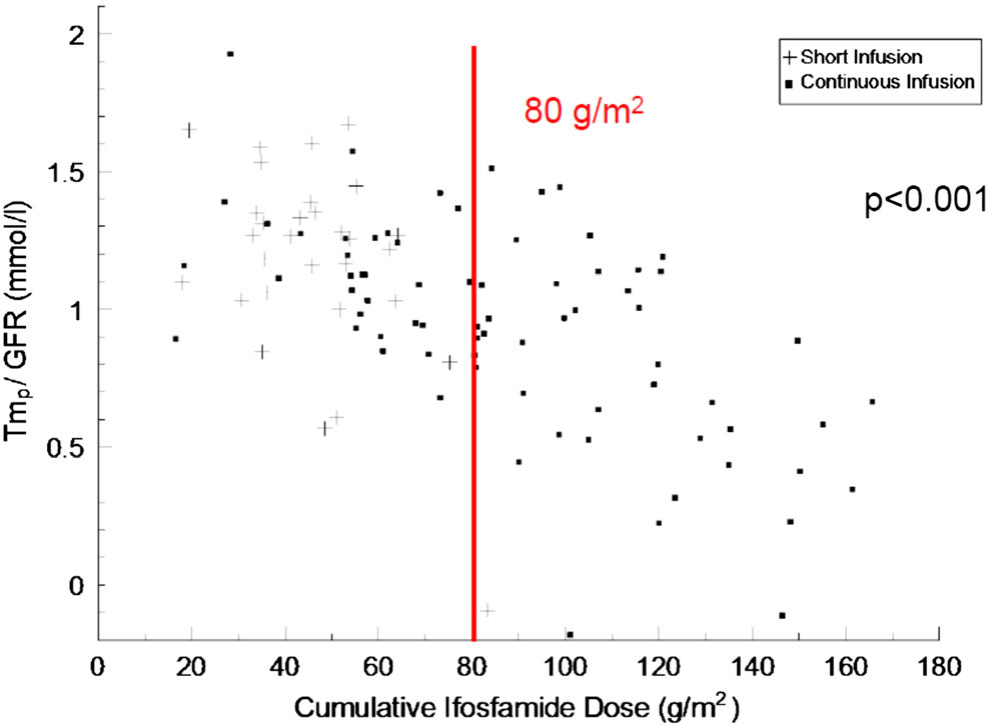
Relationship between cumulative dose of ifosfamide received and renal tubular threshold for phosphate/glomerular filtration rate (TmP/GFR) in 103 children and adolescents at a median of 6 months after ifosfamide treatment. Patients receiving ifosfamide as a short (3-h) infusion (+) or as a continuous infusion (■) are distinguished. Multiple regression analysis showed a highly significant inverse relationship between cumulative ifosfamide dose and TmP/GFR), a measure of phosphaturia severity. Severe proximal tubular toxicity (defined by hypophosphataemic rickets or myopathy, or by TmP/GFR ≤0.60 mmol/l at <12 months age or ≤0.50 mmol/l at ≥1 year age) was only observed in patients treated with higher doses of >80 g/m^2^. With permission [12]

**Fig. 2:**
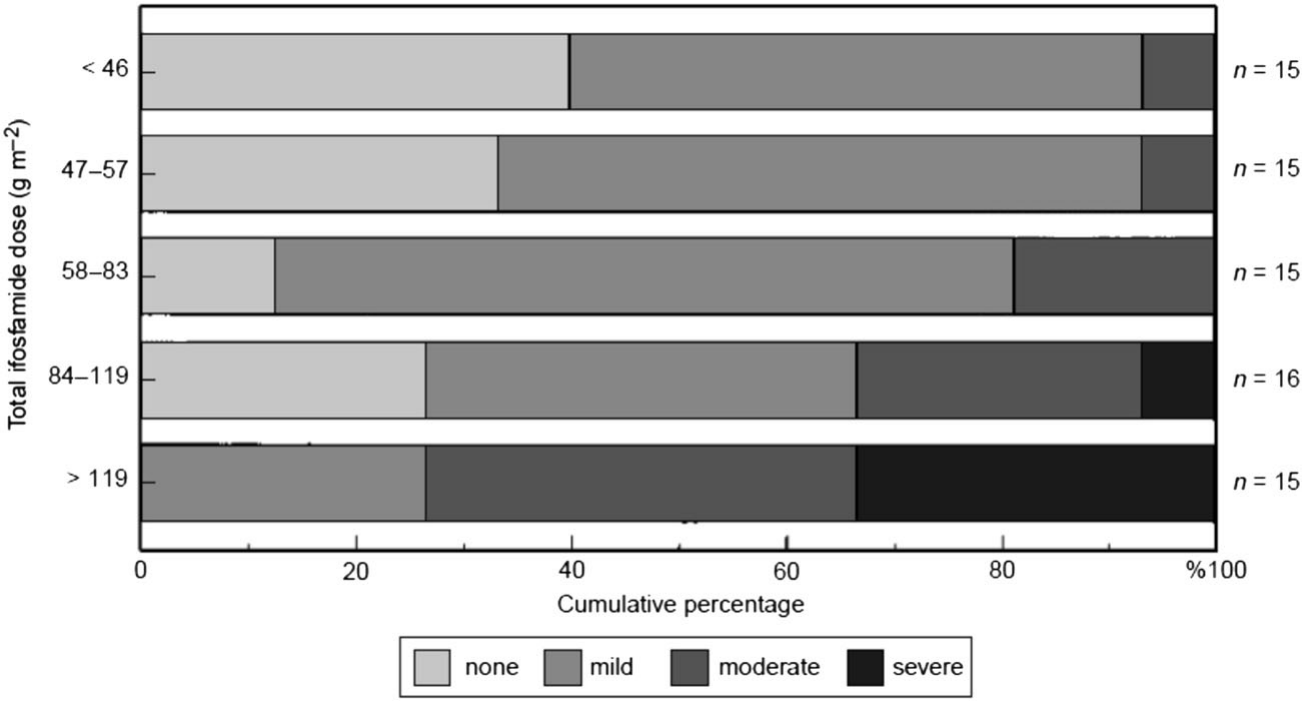
Distribution of no, mild, moderate and severe ifosfamide nephrotoxicity amongst 76 children and adolescents, assessed by calculating total nephrotoxicity score. Patients are divided into five groups according to total dose of ifosfamide received. Total nephrotoxicity score was derived from measurement and scoring of glomerular filtration rate (GFR), renal tubular threshold for phosphate (TmP)/GFR, serum bicarbonate concentration and early-morning urine osmolality. These measures give an overall evaluation of clinically important glomerular, proximal and distal nephron nephrotoxicity due to ifosfamide, reflecting those aspects of toxicity with the potential to cause morbidity or require chronic treatment. Each measure was scored on a 0–4 scale, with 0 representing no, 1 mild, 2–3 moderate and 4 severe toxicity within each individual aspect of renal damage. The individual scores are summated to give a nephrotoxicity score potentially ranging from 0 to 16. No patient receiving <84 g/m^2^ experienced severe and 20% moderate nephrotoxicity; of those receiving >119 g/m^2^, 33% experienced severe and 40% moderate nephrotoxicity. With permission [12]

A cross-sectional study of 148 patients treated with a median (range) of 62 (6–165) g/m^2^ ifosfamide at 8.1 (0.1–25) years and studied 6 (1–47) months after completion of treatment demonstrated highly significant relationships between higher ifosfamide cumulative dose and greater chronic glomerular (evaluated by radioisotope clearance GFR), proximal tubular [(serum phosphate, serum bicarbonate, renal tubular threshold for phosphate (TmP/GFR) and overall nephrotoxicity (total nephrotoxicity score; a composite of measures of glomerular, proximal and distal nephron function) [12]. However, closer inspection of data from this study illustrates the difficulty of predicting risk accurately, since there was considerable overlap between doses received by patients with and without abnormal renal function (Fig. 1). Using the total nephrotoxicity score to quantify the overall severity of nephrotoxicity, an increase in cumulative ifosfamide dose of 50 g/m^2^ increased the risk of moderate/severe toxicity nearly sevenfold. Although 7% of patients receiving ≤57 g/m^2^ ifosfamide developed moderate nephrotoxicity, severe toxicity was only observed in those who had received ≥84 g/m^2^ (Fig. 2). Multivariate analysis failed to reveal any significant independent effect of age at treatment, ifosfamide infusion schedule, or exposure to other potentially nephrotoxic agents (e.g. aminoglycosides) on nephrotoxicity.

It is important to acknowledge that risk factor analyses are necessarily limited by patient populations being studied and particularly the treatment they received. For example, in contrast to the previous study in which only three children received cisplatin and none underwent renal surgery, another cross-sectional study of 120 children and young adults included ten who underwent unilateral nephrectomy and 51 who also received cisplatin, The authors found that nephrectomised children had an 11-fold increased risk of developing Fanconi syndrome and those exposed to cisplatin a sixfold higher risk of developing phosphaturia and aminoaciduria [32]. The study also failed to find any influence of age at initial diagnosis on subsequent nephrotoxicity. Although pharmacological factors have been postulated as predictors of ifosfamide nephrotoxicity, no simple relationship has been identified between measure of ifosfamide’s pharmacokinetic or metabolic profile and either acute or chronic nephrotoxicity [36].

In summary, given our current state of knowledge, established patient- and treatment-related risk factors are unable to reliably predict all cases of significant ifosfamide nephrotoxicity.

#### Platinum

There is little information about risk factors for cisplatin nephrotoxicity in children, but high total dose and dose rate, patient age, concurrent treatment with other potential nephrotoxins and interindividual differences in cisplatin pharmacokinetics appear to be relevant. Marked glomerular (GFR) and tubular (hypomagnesaemia) toxicity was reported after a high dose rate of cisplatin (i.e. at least 40 mg/m^2^/day) in adults [38, 39], whilst higher dose rates (>40 mg/m^2^/day) were associated with greater glomerular and tubular toxicity than a lower dose rate (40 mg/m^2^/day) in children [19]. However, the influence of total dose is uncertain, with some studies in adults and children describing a relationship between cumulative dose and nephrotoxicity [23, 40] and others finding no such relationship [18, 19]. Earlier studies found no relationship between age and cisplatin nephrotoxicity in children [18, 19], but recent evidence suggests that very-long-term glomerular and tubular toxicity may be more common in children treated at an older age [41]. Although there is extensive clinical experience and some published evidence that treatment with other potential nephrotoxins, including ifosfamide, methotrexate and aminoglycosides, may exacerbate nephrotoxicity [32], and that interindividual variability in cisplatin pharmacokinetics may be important [42], there is no evidence that the risk of nephrotoxicity in clinical practice can be reduced by pharmacokinetically guided dose modification.

In contrast to cisplatin, frequency and severity of carboplatin-induced chronic hypomagnesaemia in children appears to be related to cumulative dose and older age at treatment initiation, and long-term glomerular impairment is also more common in older children [28, 41]. Since the main route of carboplatin clearance is via glomerular filtration, it is unsurprising that other potentially nephrotoxic chemotherapeutic agents (e.g. cisplatin, ifosfamide, melphalan) [43–46] and pre-existing renal dysfunction [47] may increase carboplatin-induced renal damage.

#### Relevance of risk factors in predicting nephrotoxicity

Treatment-related (especially dose characteristics) and, to a lesser extent, patient-related (e.g. age at treatment) risk factors may predict an increased risk of nephrotoxicity, but they do not predict all episodes of significant nephrotoxicity. Conversely, not all patients with risk factors develop important nephrotoxicity. Therefore, although future treatment protocols may incorporate appropriate dose ceilings for potentially nephrotoxic agents in an attempt to limit chronic renal damage (assuming there is sufficient evidence that efficacy is still maintained despite the lower doses), this strategy may still fail to protect all patients from nephrotoxicity. Careful consideration of other important risk factors is necessary, both for protocol design (e.g. caution with the use of ifosfamide in younger children, avoidance of higher cisplatin dose rates) and in individual patients (e.g. dose reduction or even avoidance of nephrotoxic agents in patients with pre-existing renal dysfunction). However, it is always important to balance the risks posed by the underlying cancer (with the potentially fatal consequences of inadequate treatment) against those of the possibility of life-changing chronic toxicity.

### Management of chronic ifosfamide- and platinum-induced nephrotoxicity

Ideally, nephrotoxicity should be minimised before chronic damage has occurred by stopping or modifying further treatment with the causative drug, but the effectiveness of this strategy is limited by the delayed onset of clinically significant renal damage in many cases, particularly of ifosfamide nephrotoxicity [11]. Furthermore, clinicians must balance the risk (for renal function) of continuing potentially damaging treatment versus the potential risk (for the likelihood of cure) of stopping it, often in the absence of proven data to guide them. Consequently, management of chronic nephrotoxicity is often supportive, aiming to prevent or ameliorate manifestations of established severe toxicity. Ifosfamide-induced tubular nephrotoxicity may necessitate prolonged supplementation with high doses of phosphate or bicarbonate to prevent HR and manifestations of RTA in growing children. Proximal RTA is difficult to correct fully, since increasing doses of bicarbonate exceed the tubular bicarbonate reabsorption threshold and are therefore simply excreted. Although 1α-hydroxy vitamin D_3_ may be beneficial in some children, this treatment carries a potential risk of metastatic calcification and nephrocalcinosis, especially in normocalcaemic patients. Magnesium supplementation may be required to prevent manifestations of severe hypomagnesaemia—such as tetany, convulsions or cardiac arrhythmias—in platinum-induced nephrotoxicity [48, 49]. Supplementation should be monitored carefully to ensure biochemical abnormalities are being corrected adequately and safely.

For the small but important subgroup of children with CKD due to severe drug-related glomerular toxicity or following extensive tumour surgery, standard renal monitoring and management should be instituted. Serial trends in serum creatinine concentration should be monitored and GFR measured when clinically indicated, although the limitations of calculated GFR based on single creatinine measurements in this patient group should be recognised [50]. Blood pressure and urine protein should be monitored regularly, since the pace of progressive glomerular impairment may be delayed by meticulous control of hypertension and introduction of an angiotensin-converting enzyme inhibitor (ACEi) or angiotensin II blocker in survivors with significant proteinuria [51]. In end-stage renal disease (ESRD), standard renal replacement treatment strategies (dialysis or transplantation) are usually appropriate in CCS, but the feasibility of some may be limited by previous treatments and interventions (e.g. extensive abdominal surgery, difficulties in vascular access after previous central lines).

### Long-term outcomes

In recent years, the frequency, nature and severity of very-long-term nephrotoxicity in CCS treated at least 5 years previously has been investigated, providing further evidence about the predictors for such late renal outcomes. GFR was calculated with the Chronic Kidney Disease Epidemiology Collaboration (CKD-EPI) formula in a cohort of 1122 5-year CCS seen in a single long-term follow-up clinic, with longitudinal data (median of 6 GFR measurements) available in 920 survivors. Median follow-up from diagnosis was 21 years, and all survivors were at least 18 years old at study [52]. Glomerular dysfunction was defined as a GFR <90 ml/min/1.73m^2^ and potentially nephrotoxic treatment as ifosfamide, cisplatin, carboplatin, high-dose methotrexate, high-dose cyclophosphamide, radiotherapy to the kidneys or nephrectomy. In survivors previously given potentially nephrotoxic treatment, compared with survivors who had not, GFR was lower [mean 95.2; 95% confidence interval (CI) 92.2–97.9 vs 100.2; 98.1–102.3 ml/min/1.73m^2^; *p* < 0.001] and the likelihood of glomerular dysfunction was higher [mean 26.4 (20.6–33.0) vs 6.6% (4.4–9.6); *p* < 0.001] up to 35 years posttreatment. GFR continued to fall with time. The highest risks were observed with larger cumulative doses of ifosfamide and cisplatin (especially >500 mg/m^2^) and with nephrectomy (especially in survivors older at the time of nephrectomy) [52].

Another study from the same group documented the prevalence of renal dysfunction in 1442 CCS evaluated once each at a median age of 19 years and median follow-up of 12.1 years from initial diagnosis. They measured blood pressure, serum magnesium, serum phosphate and urine albumin concentrations and calculated GFR using the Schwartz (in children) or CKD-EPI (in adults) formula [53]. Overall, 28.1% of survivors had at least one abnormality, including hypertension in 14.8%, albuminuria in 14.5%, reduced GFR (<90 ml/min/1.73m^2^) in 4.5%, hypomagnesaemia in 8.8% and hypophosphataemia in 3.0%. Risk factor analysis found associations between low GFR and nephrectomy with or without nephrotoxic chemotherapy (cisplatin, carboplatin, ifosfamide) and/or radiotherapy, higher cumulative ifosfamide doses and high-dose cyclophosphamide (≥1 g/m^2^/course). In addition, hypomagnesaemia was associated with cisplatin dose and/or nephrectomy, albuminuria with ifosfamide dose and hypertension with abdominal radiotherapy. Surprisingly, no predictors of hypophosphataemia were identified, although this was measured in only 46% of at-risk patients [53].

A small number of studies examined long-term renal toxicity of specific chemotherapy agents. A multicentre cross-sectional study of 183 children and adolescents previously treated with a median ifosfamide dose of 54 g/m^2^ at median age of 9.3 (0.4–27.2) years and studied once at a follow-up of 10 (5–20.7) years, found a reduced GFR (<90 ml/min/1.73m^2^) in 21% related to older age at treatment and longer duration of follow-up. Tm_p_/GFR was reduced in 24%, but only 1% were hypophosphataemic. Increased tubular phosphate loss was related to higher cumulative ifosfamide dose (*p* = 0.02) and longer duration of follow-up (*p* = 0.0005); of these factors, ifosfamide dose had the larger effect on Tm_p_/GFR. Proteinuria was observed in 12% [35].

Two smaller studies provided longitudinal data to evaluate changes in renal function over prolonged follow-up after potentially nephrotoxic chemotherapy. Both studied children and adolescents at the end of treatment and 1 and 10 years later. The first study evaluated 27 patients given a median cisplatin dose of 500 mg/m^2^ and revealed marked interindividual variability over the 10 years of follow-up. However, there was no significant change in frequency of reduced GFR (<90 ml/min/1.73m^2^) and hypomagnesaemia over the follow-up period. Lower GFR at 10 years was related to older age at treatment [41]. The second study assessed 25 patients given a median of 106 g/m^2^ ifosfamide, again showing considerable interindividual variability. However, more patients had a low GFR at 1 (72%) and 10 years (50%) years than at the end of treatment (26%) (*p* = 0.006). In contrast, clinically significant tubular toxicity present at the end of treatment resolved in all patients 10 years later. Neither dose nor age at treatment influenced the outcomes at 10 years [15].

In conclusion, chronic nephrotoxicity persists and may indeed deteriorate in many CCS, although partial or complete recovery are also well documented. Nevertheless, the occurrence of chronic glomerular dysfunction, proteinuria and hypertension is worrying in view of their potential impact on long-term health. The variable outcomes for tubular dysfunction are intriguing, with improvement usually seen after ifosfamide in contrast to persistence in many survivors who received cisplatin.

## What do we think we know?

### Guidelines and recommendations

Surveillance for late adverse effects is an increasingly important part of long-term follow-up of CCS and has been informed by the publication of long-term follow-up clinical practice guidelines by several national organisations. The International Late Effects of Childhood Cancer Guideline Harmonisation Group (IGHG) is using a rigorous evidence-based methodology to develop harmonised guidelines for surveillance to facilitate early detection of late effects [54], and renal toxicity is recognised as a high-priority topic. Whilst not yet available, it is anticipated that harmonised nephrotoxicity surveillance guidelines will be completed within the next few years.

The Dutch (LATER), UK (UKCCSG) and US (COG) guidelines [55–57] agree that high-risk patients may be defined as those who received ifosfamide, platinum drugs, renal radiotherapy including total body irradiation (TBI) or nephrectomy, but they are not as clear about the importance of particular chemotherapy-conditioning regimens for haemopoietic stem cell transplant as risk factors. They all recommend surveillance for both glomerular and tubular impairment, including measurement of serum creatinine, electrolytes, magnesium (if the patient received a platinum drug), phosphate and bicarbonate (for recipients of ifosfamide), as well as more general measures, including urinalysis (for proteinuria) and blood pressure measurement.

However, the efficacy of surveillance following these recommendations is unproven. A recent study reported 370 CCS who had undergone at least one annual long-term follow-up evaluation (total 1188). Survivors of the full range of childhood malignancies were included, with a median age of 23.9 years at first evaluation and median follow-up from malignancy diagnosis of 10.5 years. The calculated yield of positive results (percentage of positives in at-risk previously undiagnosed patients) was disappointing, being <1% for glomerular surveillance tests (urinalysis and urea/creatinine) and only 2.4% for renal tubular tests (at least two out of hypokalaemia, hypomagnesaemia and hypophosphataemia) [58]. This low yield may reflect a low rate of significant chronic nephrotoxicity in the study population but it also highlights the paucity of evidence regarding the best surveillance tests and thresholds for nephrotoxicity in CCS. Furthermore, the implications of detection or nondetection of positive results remains unclear. Although the IGHG renal surveillance guidelines, when available, will hopefully clarify some of these uncertainties, these findings illustrate the difficulties of designing effective surveillance strategies that will detect potentially treatable late effects in a timely manner capable of improving health outcomes.

### Pathogenesis

Ifosfamide nephrotoxicity is assumed to be caused by a toxic metabolite produced in significant amounts in the kidney by the breakdown of ifosfamide but not that of cyclophosphamide. Animal models of renal tubular cell culture suggest that the mechanism involves cellular oxidative stress leading to mitochondrial damage and energy depletion [59]. Chloroacetaldehyde has been implicated as a potential candidate, with the hypothesis that quantitative differences in its production may account for the great variability in renal outcomes in patients exposed to ifosfamide; some individuals experience considerable toxicity with relatively small cumulative doses, others appear to experience no adverse effects despite large doses [60]. However, chloroacetaldehyde is not yet proven conclusively to be primarily or solely responsible for ifosfamide nephrotoxicity, and uncertainty remains about the cellular and molecular mechanisms of damage.

The identity of the agent responsible for platinum nephrotoxicity is even less clear. The differential nephrotoxicity of cisplatin and carboplatin affords a clue by suggesting that the greater frequency and severity of toxicity after cisplatin results from formation of increased amounts of a putative nephrotoxic metabolite due to increased lability of the chloride ligand of cisplatin compared with the cyclobutene dicarboxylate group of carboplatin. Many mechanisms of platinum nephrotoxicity have been postulated, including direct cellular toxic, vasoconstrictive and proinflammatory effects [61]. Several protective agents have been suggested or investigated corresponding to these mechanisms, most often in animal models or small clinical pilot studies [62]. Amifostine is an organic thiophosphate prodrug hydrolysed in vivo by alkaline phosphatase to an active cytoprotectant thiol compound, WR-1065, which protects healthy cells preferentially to malignant cells. It reduced nephrotoxicity in a randomised clinical trial in women receiving cisplatin for ovarian cancer [63], and American Society of Clinical Oncology (ASCO) guidelines recommend its use be considered in patients receiving cisplatin [64]. However, no protective agents have yet demonstrated convincing benefit in children, and none has entered routine clinical practice in paediatric malignancies.

Uncertainty about the primary pathogenesis of ifosfamide- and platinum-induced nephrotoxicity is particularly relevant since tubular injury is a prominent component in both, and very important in light of the increasing recognition that proximal tubular injury is an important driver of subsequent progressive CKD [65, 66].

## What are we still learning?

### How common is very-long-term nephrotoxicity and what are its implications?

There are many aspects of nephrotoxicity about which much remains to be learned. It is important to recognise that vigilance is required, since the nephrotoxicity of ifosfamide was not predicted by preclinical studies, and since apparently normal renal function on completion of treatment does not necessarily exclude the later development of significant nephrotoxicity. Indeed, chronic renal impairment may not become evident until months or years later, as shown by the often delayed onset of nitrosourea nephrotoxicity [67], highlighting the importance of long-term follow-up studies.

Despite clinical experience of long-term renal toxicity in a small number of individual children due to other treatments, such as high-dose methotrexate, renal radiotherapy and surgery—and, in the case of nephrectomy, confirmation of its importance in causing CKD (stage ≥2) in a large cohort study [52]—there is much less published information about the clinical nature and long-term outcome of nephrotoxicity due to these treatments. Indeed, large cohort studies have failed to demonstrate a significant association between high-dose methotrexate and chronic nephrotoxicity in CCS [52, 68]. Similarly, notwithstanding the recent evidence about outcomes 5–20 years after treatment, there is still very little published information about the prevalence and nature of nephrotoxicity at later time points. This is particularly important given the expected decline in renal function that occurs as part of the natural ageing process and the recent observation that many CCS display evidence of an accelerated ageing phenotype manifest by frailty [69]. It is likely that the reduction of physiological reserve implied by this process will include renal function and will interact adversely with co-existent chronic nephrotoxicity, potentially leading to an increased risk of clinically significant renal impairment in middle-aged and older survivors.

There is increasing recognition of the acute nephrotoxicity of new, targeted, anticancer drugs, which is derived predominantly from adult studies [70]; however, insufficient data and follow-up duration is available to evaluate long-term outcomes. Recent data has highlighted the occurrence of AKI with histological features of acute tubulointerstitial nephritis in up to 2% of adults treated with immune checkpoint inhibitors, although corticosteroids led to partial improvement in most patients [71, 72]. There is also increasing recognition of minimal change/focal segmental glomerulosclerosis and TMA in patients treated with vascular endothelial growth factor (VEGF) inhibitors [73]. Again, these glomerular disorders appear to be reversible with discontinuation of the causative agents. Nevertheless, in view of the risk of significant renal damage, which may have potentially severe and lasting consequences, active surveillance has been recommended in patients treated with immune checkpoint inhibitors [74]. In a broader sense, the emerging field of onconephrology reflects the importance of studying adverse renal outcomes in patients treated with new anticancer agents to increase our understanding of the causes and natural history of AKI and CKD in the growing population of cancer survivors of all ages, as well as providing information for individual patient management [73–75].

### Can we predict or prevent chronic nephrotoxicity?

Accurate prediction of the likelihood and severity of renal toxicity after treatment with known nephrotoxic agents is not yet feasible despite considerable study of potential risk factors. Further research is required to examine other potential confounding and causative factors, including host factors such as genetic polymorphisms, baseline renal function and treatment-related pharmacokinetic variables.

Treatment-induced nephrotoxicity may be prevented or reduced by general or specific strategies. General approaches to the use of potentially nephrotoxic agents, based on our admittedly incomplete knowledge of risk factors, may include carefully considered treatment adjustments, such as dose limitation where the increased toxicity of higher doses is clear (e.g. for ifosfamide and radiotherapy) [3, 12], or subtotal rather than total nephrectomy (nephron-sparing surgery) [76]. For drug-induced nephrotoxicity, hyperhydration is used with most cisplatin, ifosfamide and methotrexate regimens to reduce renal accumulation of toxic metabolites, whilst some cisplatin administration schedules also employ mannitol diuresis, although there is little clear evidence that this reduces nephrotoxicity [77].

Ideally, nephrotoxicity will be reduced or—hopefully—eliminated by the development of nontoxic or less toxic agents, but this is likely to require improved understanding of the detailed pathogenesis of renal damage.

#### Key summary points

1. Nephrotoxicity is an important long-term risk for CCS.
2. Many disease and treatment-related causes, including ifosfamide, cisplatin, carboplatin, radiotherapy involving the kidneys and nephrectomy, may contribute individually or collectively to renal toxicity.
3. Ifosfamide, cisplatin and carboplatin may all cause glomerular or renal tubular toxicity, or both, although the clinical manifestations of tubulopathy differ between ifosfamide (ranging from hypophosphataemia to a Fanconi syndrome) and the platinum agents (typically hypomagnesaemia).
4. Currently recognised risk factors do not predict all episodes of nephrotoxicity.
5. Improved understanding of the pathogenesis of nephrotoxicity is vital to reduce the frequency and severity of nephrotoxicity.

#### Multiple-choice questions (answers are provided following the reference list)

1. Which of the following statements about chronic nephrotoxicity is correct?

- The most common adverse effect of nephrectomy is tubular dysfunction.
- Radiotherapy exposing the kidneys may cause hypertension years after treatment.
- Ifosfamide causes hypomagnesaemia.
- The commonest manifestation of the tubular toxicity of platinum drugs is the Fanconi syndrome.
- Acute kidney injury usually leads to later tubular toxicity.

2. The most common presentation of chronic ifosfamide nephrotoxicity in children is:

- Acute kidney injury.
- Hypomagnesaemia.
- Hypophosphataemia.
- Hypocalcaemia.
- Haematuria.

3. Which of the following statements about prevention or prediction of chronic nephrotoxicity is correct?

- Mesna prevents cisplatin nephrotoxicity.
- Children with ifosfamide-induced hypophosphataemia should always be switched from ifosfamide to cyclophosphamide.
- Mannitol prevents ifosfamide nephrotoxicity.
- The use of new drugs in which preclinical studies have not demonstrated renal toxicity will eliminate the risk of chronic nephrotoxicity.
- Absence of renal toxicity at the end of anticancer treatment does not exclude future chronic nephrotoxicity.

4. Platinum drug nephrotoxicity in children:

- Is more common after carboplatin than after cisplatin
- Recovers by 10 years after treatment
- Randomised controlled trial evidence shows it can be prevented by amifostine
- Can lead to cardiac arrhythmias
- Is commoner in infants than in older children

5. Which of the following statements about management of chronic nephrotoxicity is correct?

- Renal transplantation for ESRD is contraindicated in CCS.
- Magnesium supplements may be required to treat or prevent complications of platinum drug nephrotoxicity.
- Vitamin D should be avoided in children with ifosfamide nephrotoxicity.
- Hypertension is to be expected in patients with cisplatin nephrotoxicity and does not need to be treated.
- GFR should be measured (not calculated) every year in CCS.

## References

[1] Oeffinger KC, Mertens AC, Sklar CA, Kawashima T, Hudson MM, Meadows AT, Friedman DL, Marina N, Hobbie W, Kadan-Lottick NS, Schwartz CL, Leisenring W, Robison LL (2006). Chronic health conditions in adult survivors of childhood cancer. N Engl J Med.

[2] Skinner R (2010). Nephrotoxicity of cancer treatment in children. Pediatr Health.

[3] Dawson LA, Kavanagh BD, Paulino AC, Das SK, Miften M, Li XA, Pan C, Ten Haken RK, Schultheiss TE (2010). Radiation-associated kidney injury. Int J Radiation Oncology Biol Phys.

[4] Luxton RW (1961). Radiation nephritis. Lancet.

[5] Donckerwolke RM, Coppes MJ (2001). Adaptation of renal function after unilateral nephrectomy in children with renal tumors. Pediatr Nephrol.

[6] Welch TR, McAdams AJ (1986). Focal glomerulosclerosis as a late sequela of Wilms tumor. J Pediatr.

[7] Schiavetti A, Altavista P, De Luca L, Andreoli G, Megaro G, Versacci P (2015). Long-term renal function in unilateral non-syndromic renal tumor survivors treated according to International Society of Pediatric Oncology protocols. Pediatr Blood Cancer.

[8] Willemse PHB, de Jong PE, Elema JD, Mulder NH (1989). Severe renal failure following high-dose ifosfamide and mesna. Cancer Chemother Pharmacol.

[9] Friedlander MM, Haviv YS, Rosenmann E, Peylan-Ramu N (1998). End-stage renal interstitial fibrosis in an adult ten years after ifosfamide therapy. Am J Nephrol.

[10] Loebstein R (1999). Risk factors for long-term outcome of ifosfamide-induced nephrotoxicity in children. J Clin Pharmacol.

[11] Prasad VK, Lewis IJ, Aparicio SR, Heney D, Hale JP, Bailey CC, Kinsey SE (1996). Progressive glomerular toxicity of ifosfamide in children. Med Pediatr Oncol.

[12] Skinner R, Cotterill S, Stevens MCG (2000). Risk factors for nephrotoxicity after ifosfamide treatment in children: a UKCCSG late effects group study. Br J Cancer.

[13] Skinner R, Pearson ADJ, English MW, Price L, Wyllie RA, Coulthard MG, Craft AW (1996). Risk factors for ifosfamide nephrotoxicity in children. Lancet.

[14] Church DN, Hassan AB, Harper SJ, Wakeley CJ, Price CGA (2007). Osteomalacia as a late metabolic complication of ifosfamide chemotherapy in young adults: illustrative cases and review of the literature. Sarcoma.

[15] Skinner R, Parry A, Price L, Cole M, Craft AW, Pearson ADJ (2010). Glomerular toxicity persists ten years after ifosfamide treatment in childhood and is not predictable by age or dose. Pediatr Blood Cancer.

[16] Farry JK, Flombaum CD, Latcha S (2012). Long term renal toxicity of ifosfamide in adult patients – 5 year data. Eur J Cancer.

[17] Stöhr W, Patzer L, Paulides M, Kremers A, Beck JD, Langer T, Rossi R (2007). Growth impairment after ifosfamide-induced nephrotoxicity in children. Pediatr Blood Cancer.

[18] Brock PR, Koliouskas DE, Barratt TM, Yeomans E, Pritchard J (1991). Partial reversibility of cisplatin nephrotoxicity in children. J Pediatr.

[19] Skinner R, Pearson ADJ, English MW, Price L, Wyllie RA, Coulthard MG, Craft AW (1998). Cisplatin dose rate as a risk factor for nephrotoxicity in children. Br J Cancer.

[20] Womer RB, Pritchard J, Barratt TM (1985). Renal toxicity of cisplatin in children. J Pediatr.

[21] Stöhr W, Paulides M, Bielack S, Jurgens H, Koscielniak E, Rossi R, Langer T, Beck JD (2007). Nephrotoxicity of cisplatin and carboplatin in sarcoma patients: a report from the late effects surveillance system. Pediatr Blood Cancer.

[22] Goren MP (2003). Cisplatin nephrotoxicity affects magnesium and calcium metabolism. Med Pediatr Oncol.

[23] Bianchetti MG, Kanaka C, Ridolfi-Luthy A, Wagner HP, Hirt A, Paunier L, Peheim E, Oetliker OH (1990). Chronic renal magnesium loss, hypocalciuria and mild hypokalaemic metabolic alkalosis after cisplatin. Pediatr Nephrol.

[24] Canpolat C, Pearson P, Jaffe N (1994). Cisplatin-associated hemolytic uremic syndrome. Cancer.

[25] Blake-Haskins JA, Lechleider RJ, Kreitman RJ (2011). Thrombotic microangiopathy with targeted cancer agents. Clin Cancer Res.

[26] Latcha S, Jaimes EA, Patil S, Glezerman IG, Mehta S, Flombaum CD (2016). Long-term renal outcomes after cisplatin treatment. Clin J Am Soc Nephrol.

[27] Harrell RM, Sibley R, Vogelzang NJ (1982). Renal vascular lesions after chemotherapy with vinblastine, bleomycin and cisplatin. Am J Med.

[28] English MW, Skinner R, Pearson ADJ, Price L, Wyllie R, Craft AW (1999). Dose-related nephrotoxicity of carboplatin in children. Br J Cancer.

[29] Craft AW, Pearson ADJ (1989). Three decades of chemotherapy for childhood cancer: from cure ‘at any cost’ to cure ‘at least cost’. Cancer Surv.

[30] Armenian SH, Landier W, Hudson MM, Robison LL, Bhatia S (2013). Children’s Oncology Group’s 2013 blueprint for research: survivorship and outcomes. Pediatr Blood Cancer.

[31] Raney B, Ensign LG, Foreman J, Khan F, Newton W, Ortega J, Ragab A, Wharam M, Wiener E, Maurer H (1994). Renal toxicity of ifosfamide in pilot regimens of the intergroup rhabdomyosarcoma study for patients with gross residual disease. Am J Pediatr Hematol Oncol.

[32] Rossi R, Godde A, Kleinebrand A, Riepenhausen M, Boos J, Ritter J, Jurgens H (1994). Unilateral nephrectomy and cisplatin as risk factors of ifosfamide-induced nephrotoxicity: analysis of 120 patients. J Clin Oncol.

[33] Shore R, Greenberg M, Geary D, Koren G (1992). Iphosphamide-induced nephrotoxicity in children. Pediatr Nephrol.

[34] Stöhr W, Paulides M, Bielack S, Jurgens H, Treuner J, Rossi R, Langer T, Beck JD (2007). Ifosfamide-induced nephrotoxicity in 593 sarcoma patients: a report from the late effects surveillance system. Pediatr Blood Cancer.

[35] Oberlin O, Fawaz O, Rey A, Niaudet P, Ridola V, Orbach D, Bergeron C, Defachelles AS, Gentet JC, Schmitt C, Rubie H, Munzer M, Plantaz D, Deville A, Minard V, Corradini N, Leverger G, de Vathaire F (2009). Long-term evaluation of ifosfamide-related nephrotoxicity in children. J Clin Oncol.

[36] Boddy AV, English MW, Pearson ADJ, Idle JR, Skinner R (1996). Ifosfamide nephrotoxicity: limited influence of metabolism and mode of administration during repeated therapy in paediatrics. Eur J Cancer.

[37] Le Deley MC, Paulussen M, Lewis I, Brennan B, Ranft A, Whelan J, Le Teuff G, Michon J, Ladenstein R, Marec-Berard P, van den Berg H, Hjorth L, Wheatley K, Judson I, Juergens H, Craft A, Oberlin O, Dirksen U (2014). Cyclophosphamide compared with ifosfamide in consolidation treatment of standard-risk Ewing sarcoma: results of the randomized noninferiority euro-EWING99-R1 trial. J Clin Oncol.

[38] Daugaard G, Abildgaard U, Holsten-Rathlou NH, Bruunshuus I, Bucher D, Leyssac PP (1988). Renal tubular function in patients treated with high-dose cisplatin. Clin Pharmacol Ther.

[39] Daugaard G, Rossing N, Rorth M (1988). Effects of cisplatin on different measures of glomerular function in the human kidney with special emphasis on high-dose. Cancer Chemother Pharmacol.

[40] Lam M, Adelstein DJ (1986). Hypomagnesemia and renal magnesium wasting in patients treated with cisplatin. Am J Kidney Dis.

[41] Skinner R, Parry A, Price L, Cole M, Craft AW, Pearson ADJ (2009). Persistent nephrotoxicity during ten year follow-up after cisplatin or carboplatin treatment in childhood: relevance of age and dose as risk factors. Eur J Cancer.

[42] Reece PA, Stafford I, Russell J, Khan M, Gill PG (1987). Creatinine clearance as a predictor of ultrafilterable platinum disposition in cancer patients treated with cisplatin: relationship between peak ultrafilterable platinum plasma levels and nephrotoxicity. J Clin Oncol.

[43] Curt GA, Grygiel JJ, Corden BJ, Ozols RF, Weiss RB, Tell DT, Myers CE, Collins JM (1983). A phase I and pharmacokinetic study of diaminecyclobutane-dicarboxylatoplatinum (NSC 241240). Cancer Res.

[44] Frenkel J, Kool G, de Kraker J (1995). Acute renal failure in high dose carboplatin chemotherapy. Med Pediatr Oncol.

[45] Gordon SJ, Pearson AD, Reid MM, Craft AW (1992). Toxicity of single-day high-dose vincristine, melphalan, etoposide and carboplatin consolidation with autologous bone marrow rescue in advanced neuroblastoma. Eur J Cancer.

[46] McDonald BR, Kirmani S, Vasquez M, Mehta RL (1991). Acute renal failure associated with the use of intraperitoneal carboplatin: a report of two cases and review of the literature. Am J Med.

[47] Foster BJ, Clagett-Carr K, Leyland-Jones B, Hoth D (1985). Results of NCI-sponsored phase I trials with carboplatin. Cancer Treat Rev.

[48] Bano N, Najam R, Qazi F (2013). Adverse cardiac manifestations of cisplatin - a review. Int J Pharm Sci Rev Res.

[49] Bellin SL, Selim M (1988). Cisplatin-induced hypomagnesemia with siezures: a case report and review of the literature. Gynecol Oncol.

[50] Skinner R, Cole M, Pearson ADJ, Keir MJ, Price L, Wylie RA, Coulthard MG, Craft AW (1994). Inaccuracy of glomerular filtration rate estimation from height/plasma creatinine ratio. Arch Dis Child.

[51] Bardi E, Olah AV, Bartyik K, Endreffy E, Jenei C, Kappelmayer J, Kiss C (2004). Late effects on renal glomerular and tubular function in childhood cancer survivors. Pediatr Blood Cancer.

[52] Mulder RL, Knijnenburg SL, Geskus RB, van Dalen EC, van der Pal HJH, Koning CCE, Bouts AH, Caron HN, Kremer LCM (2013). Glomerular function time trends in long-term survivors of childhood cancer: a longitudinal study. Cancer Epidemiol Biomark Prev.

[53] Knijnenburg SL, Jaspers MW, van der Pal HJ, Schouten-van Meeteren AY, Bouts AH, Lieverst JA, Bokenkamp A, Koning CCE, Oldenburger F, Wilde JCH, van Leeuwen FE, Caron HN, Kremer LC (2012). Renal dysfunction and elevated blood pressure in long-term childhood cancer survivors. Clin J Am Soc Nephrol.

[54] Kremer LC, Mulder RL, Oeffinger KC, Bhatia S, Landier W, Levitt G, Constine LS, Wallace WH, Caron HB, Skinner R, Hudson MM (2013). A worldwide collaboration to harmonize guidelines for the long-term follow-up of childhood cancer survivors: a report from the International late effects of childhood cancer Guideline harmonization group. Pediatr Blood Cancer.

[55] Dutch Childhood Oncology Group (2010) Richtlijn follow-up na kinderkanker meer dan 5 jaar na diagnose. SKION. http://www.skion.nl/. Accessed 9 May 2016

[56] Children’s Oncology Group (COG) (2013) Long-term follow-up guidelines for survivors of childhood, adolescent, and young adult cancers. http://www.survivorshipguidelines.org/. Accessed 9 May 2016

[57] Skinner R, Wallace WHB, Levitt GA (2005) United Kingdom Children’s Cancer Study Group Late Effects Group. Therapy based long term follow up practice statement. United Kingdom Children’s Cancer Study Group. http://www.cclg.org.uk/write/MediaUploads/Member%20area/Treatment%20guidelines/LTFU-full.pdf. Accessed 9 May 2016

[58] Landier W, Armenian SH, Lee J, Thomas O, Wong FL, Francisco L, Herrera C, Kasper C, Wilson KD, Zomorodi M, Bhatia S (2012). Yield of screening for long-term complications using the Children’s Oncology group long-term follow-up guidelines. J Clin Oncol.

[59] Nissim I, Horyn O, Daikhin Y, Nissim I, Luhovyy B, Phillips PC, Yudkoff M (2006). Ifosfamide-induced nephrotoxicity: mechanism and prevention. Cancer Res.

[60] Skinner R, Sharkey IM, Pearson ADJ, Craft AW (1993). Ifosfamide, mesna, and nephrotoxicity in children. J Clin Oncol.

[61] Miller RP, Tadagavadi RK, Ramesh G, Reeves WB (2010). Mechanisms of cisplatin nephrotoxicity. Toxins.

[62] Pinzani V, Bressolle F, Haug IJ, Galtier M, Blayac JP, Balmes P (1994). Cisplatin-induced renal toxicity and toxicity-modulating strategies: a review. Cancer Chemother Pharmacol.

[63] Kemp G, Rose P, Lurain J, Berman M, Manetta A, Roullet B, Homesley H, Belpomme D, Glick J (1996). Amifostine pretreatment for protection against cyclophosphamide-induced and cisplatin-induced toxicities: results of a randomized control trial in patients with advanced ovarian cancer. J Clin Oncol.

[64] Hensley ML, Hagerty KL, Kewalramani T, Green DM, Meropol NJ, Wasserman TH, Cohen GI, Emami B, Gradishar WJ, Mitchell RB, Thigpen JT, Trotti A, von Hoff D, Schuchter LM (2009). American Society of Clinical Oncology 2008 clinical practice Guideline update: use of chemotherapy and radiation therapy protectants. J Clin Oncol.

[65] Chevalier RL (2016). The proximal tubule is the priomary target of injury and progression of kidney disease: role of the glomerulotubular junction. Am J Physiol Renal Physiol.

[66] Takaori K, Yanahgita M (2016). Insights into the mechanisms of the acute kidney injury-to-chronic kidney disease continuum. Nephron.

[67] Harman WE, Cohen HJ, Schneeberger EE, Grupe WE (1979). Chronic renal failure in children treated with methyl CCNU. N Engl J Med.

[68] Dekkers IA, Blijdorp K, Carnsberg K, Pluijm SM, Pieters R, Neggers SJ, van den Heuvel-Eibrink MM (2013). Long-term nephrotoxicity in adult survivors of childhood cancer. Clin J Am Soc Nephrol.

[69] Ness KK, Krull KR, Jones KE, Mulrooney DA, Armstrong GT, Green DM, Chemaitilly W, Smith WA, Wilson CL, Sklar CA, Shelton K, Srivastava DK, Ali S, Robison LL, Hudson MM (2013). Physiologic frailty as a sign of accelerated aging among adult survivors of childhood cancer: a report from the St Jude lifetime cohort study. J Clin Oncol.

[70] Porta C, Cosmai L, Gallieni M, Pedrazzoli P, Malberti F (2015). Renal effects of targeted anticancer therapies. Nat Rev Nephrol.

[71] Belliere J, Meyer N, Mazieres J, Ollier S, Boulinguez S, Delas A, Ribes D, Faguer S (2016). Acute interstitial nephritis related to immune checkpoint inhibitors. Br J Cancer.

[72] Cortazar FB, Marrone KA, Troxell ML, Ralto KM, Hoenig MP, Brahmer JR, Le DT, Lipson EJ, Glezerman IG, Wolchok J, Cornell LD, Feldman P, Stokes MB, Zapata SA, Hodi FS, Ott PA, Yamashita M, Leaf DE (2016). Clinicopathological features of acute kidney injury associated with immune checkpoint inhibitors. Kidney Int.

[73] Ollero M, Sahali D (2014). Inhibition of the VEGF signalling pathway and glomerular disorders. Nephrol Dial Transplant.

[74] Perazella MA (2016). Checkmate: kidney injury associated with targeted cancer immunotherapy. Kidney Int.

[75] Finkel KW, Howard SC (2014). Onco-nephrology: an invitation to a new field. J Clin Oncol.

[76] Cozzi F, Schiavetti A, Morini F, Zani A, Gambino M, Donfrancesco C, Cozzi DA (2005). Renal function adaptation in children with unilateral renal tumors treated with nephron sparing surgery or nephrectomy. J Urol.

[77] Ruggiero A, Rizzo D, Trombatore G, Maurizi P, Riccardi R (2016). The ability of mannitol to decrease cisplatin-induced nephrotoxicity in children: real or not?. Cancer Chemother Pharmacol.

